# Use of the Illumina EPIC methylation array for epigenomic research in the crab‐eating macaque (*Macaca fascicularis*)

**DOI:** 10.1002/npr2.12145

**Published:** 2020-10-10

**Authors:** Yutaka Nakachi, Kazuhiro Ishii, Miki Bundo, Tomoyuki Masuda, Kazuya Iwamoto

**Affiliations:** ^1^ Department of Molecular Brain Science Graduate School of Medical Sciences Kumamoto University Kumamoto Japan; ^2^ Department of Neurology Faculty of Medicine University of Tsukuba Ibaraki Japan

**Keywords:** bisulfite modification, cynomolgus monkey, cytosine, DNA methylation, nonhuman primate

## Abstract

**Background:**

Commercially available Illumina DNA methylation arrays (HumanMethylation 27K, HumanMethylation450, and MethylationEPIC BeadChip) can be used for comprehensive DNA methylation analyses of not only the human genome but also other mammalian genomes, ranging from those of nonhuman primates to those of rodents. However, practical application of the EPIC array to the crab‐eating macaque has not been reported.

**Methods:**

Through bioinformatic analyses involving cross‐species comparison and consideration of probe performance, we selected array probes that can be reliably used for the crab‐eating macaque genome. A DNA methylation assay using an EPIC array was performed on genomic DNA extracted from the brains of five crab‐eating macaques. The obtained DNA methylation data were compared with a publicly available dataset.

**Results:**

Among the 865 918 probes in the EPIC array, a total of 183 509 probes (21.2%) were selected as high‐confidence array probes in the crab‐eating macaque. Subsequent comparisons revealed that the data from these probes showed good concordance with other DNA methylation datasets of the crab‐eating macaque.

**Conclusion:**

The selected high‐confidence array probes would be useful for high‐throughput DNA methylation assays of the crab‐eating macaque.

## Abstract 2

Epigenetic research in the non‐human primates, such as crab‐eating macaque, will be important to understand the pathophysiology of psychiatric disorders. Among the methylation array probes for human genome, the probes that can reliably measure DNA methylation levels of the crab‐eating macaque are reported.

## Graphical abstract

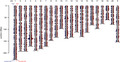

## 1 INTRODUCTION

DNA methylation is a central epigenetic mechanism that regulates long‐lasting gene expression and is considered to reflect the complex interaction between genes and environments. Altered DNA methylation in the brain has been reported in a wide range of neuropsychiatric disorders and their animal models. Thus, elucidating the comprehensive DNA methylation profile may be quite important for understanding pathophysiology.^1^, ^2^

The commercially available array‐based DNA methylation assay, which allows quantification of DNA methylation levels at hundreds of thousands of CpG sites, has been widely used in humans. Although the assay was specifically designed for the human genome, previous studies have reported its application to the genomes of other species, ranging from nonhuman primates to rodents.^3^, ^4^, ^5^, ^6^, ^7^, ^8^, ^9^ Typically, this was done by assessing the conservation of genomic context around the probe region between humans and the species of interest by a bioinformatic approach.

The crab‐eating macaque (cynomolgus monkey, *Macaca fascicularis*) is a cercopithecine primate in the group of Old World monkeys and is widely distributed in Southeast Asia. Due to its ease of rearing and propagation, as well as the close relatedness of its brain structure and function to those of humans, this macaque has become one of the most widely used nonhuman primates in biomedical research.

Previously, applications of HumanMethylation27K and HumanMethylation450K arrays (Illumina), which contain approximately 27 000 and 450 000 array probes, respectively, to the crab‐eating macaque have been reported.^3^, ^8^ Here, we report the application of the updated version of the array, MethylationEPIC (Illumina), containing approximately 850 000 probes, to the crab‐eating macaque.

## 2 MATERIALS AND METHODS

### 2.1 Animals

Five cynomolgus monkeys (*Macaca fascicularis*; 2 males and 3 females, 3‐4 years old) were purchased from HAMRI Co., Ltd. All procedures for animal care and experimentation were approved by the University of Tsukuba Animal Experiment Committee and were carried out in accordance with the Guidelines for Proper Conduct of Animal Experiments by the Science Council of Japan.

### 2.2 Probe filtering and selection strategy

Probe selection was performed according to our previous report.^9^ In brief, we used the manifest file provided by the manufacturer (Illumina). The information for SourceSeq, ProbeSeq, MapInfo, and AlleleA ProbeSeq was provided in the manifest file (Infinium MethylationEPIC v1.0 B4), which indicated the bisulfite‐unconverted design sequence, the probe sequence, the coordinates of the target site, and the sequence for probe A, respectively. We retrieved human bisulfite‐unconverted probe sequences (referred to as modified SourceSeq) based on the MapInfo and AlleleA ProbeSeq. Illumina arrays include two types of array probes. Type I probe contains two probes designed for methylated and unmethylated CpG at one CpG site, whereas type II probe contains single probe for calculation of methylation level at one CpG site. For type I and type II probes, we retrieved 50 and 51 bp of the human sequence, respectively. We mapped the modified SourceSeq to the reference genome of the crab‐eating macaque, Macaca_fascicularis_5.0 (macFas5, GCA_000364345.1 without the mitochondrial sequence). We used Blat^10^ for mapping and selected probes with the following criteria: (a) no insertions/deletions; (b) only one best hit and no suboptimal hits; (c) no mismatches within 5 bp of the target site, with up to two mismatches allowed in the rest of the sequence; and (d) no ambiguous bases in the mapped reference genome. All location descriptions of mapped probes and the relationship of orthologous genes between humans and the crab‐eating macaque were confirmed by using the Ensembl release 96 dataset corresponding to the Macaca_fascicularis_5.0 genome. Selected probes were visualized with a custom viewer (CHARANGO viewer; CHromosomal Alignment Representation and ANnotation Graphical Omics viewer). Scripts are available upon request.

### 2.3 DNA methylation assay

Genomic DNA was extracted from frozen occipital lobes using the AllPrep DNA/RNA Mini Kit (QIAGEN). DNA methylation profiles were obtained using an Illumina MethylationEPIC kit (Illumina). All experimental methods were performed strictly according to the manufacturer's protocols (Illumina).

### 2.4 Data analysis

The IDAT file, a raw data file containing the intensities of the probes in the array, was analyzed using the *minfi* package^11^ of R/Bioconductor and noob background correction.^12^ The probes for which detection *P* values were <.01 across all samples (N = 5) were used. Pairwise Pearson's correlations of beta values, which are proxies for DNA methylation level and range from 0 to 1, were calculated. Average beta values were used for comparison with the published dataset.^13^

## 3 RESULTS AND DISCUSSION

**FIGURE 1:**
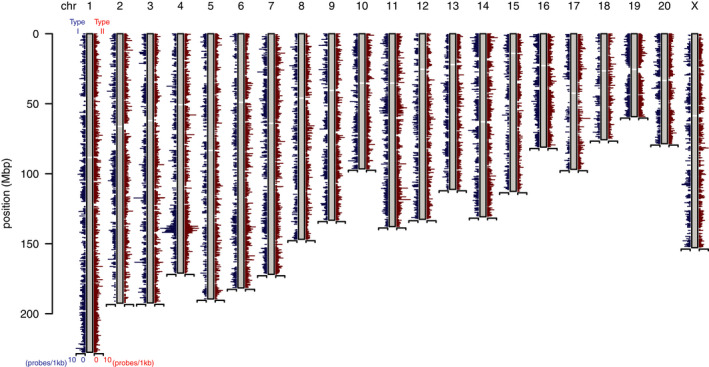
Chromosomal distribution of high‐confidence EPIC array probes. Chromosomal locations of high‐confidence probes (N = 183 509) are shown. Both type I (N = 37 214, left‐hand side of each chromosome) and type II (N = 146 295, right‐hand side of each chromosome) probes are illustrated

Among the 865 918 probes in the HumanMethylationEPIC array, we selected the probes that could hybridize with the bisulfite‐converted crab‐eating macaque genome (macFas5). As a result, we obtained 183 509 high‐confidence probes in total (Figure 1, Table S1). These probes account for 21.2% of all the probes in the array. In our similar calculations in the common marmoset^9^ and the chimpanzee (Nakachi et al, unpublished data), we obtained approximately 9% and 44% high‐confidence probes, respectively. Considering the evolutionary relationships, the number of obtained probes in this study seems reasonable. We added annotation based on macFas5 to each probe, by which one can consider orthologous relationships between humans and the crab‐eating macaque (Table S1).

The high‐confidence probes were mapped to the entire macaque genome for both type I (N = 37 214) and type II (N = 146 295) probes (Figure 1). To validate the performance of the high‐confidence probes, we profiled DNA methylation patterns of the crab‐eating macaque brains using the EPIC array (occipital lobes, N = 5). In this analysis, we used autosomal CpG probes (N = 174 611) that passed detection *P* values (<.01) across five samples. The finally selected probes (N = 172 688) showed high concordance not only for all probes (total average *R* = 0.980) but also for both type I (*R* = 0.989) and type II (*R* = 0.974) probes among five biological replicates.

**FIGURE 2:**
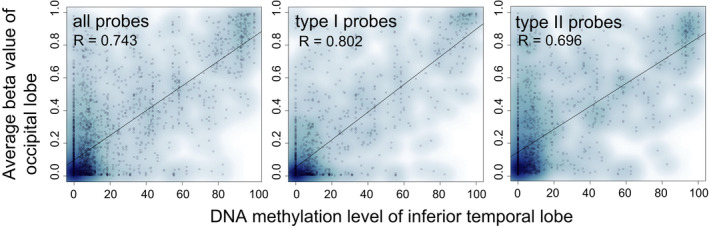
Comparison of DNA methylation levels between high‐confidence probes and other datasets. Average beta values (N = 5) of high‐confidence probes and methylation levels reported in other datasets^13^ are plotted. Among the comparisons with seven different brain regions, data on the inferior temporal lobe are shown as an example

We then compared the DNA methylation values of high‐confidence probes and independent DNA methylation datasets for the crab‐eating macaque. Because the DNA methylation study using the occipital lobe of the crab‐eating macaque has not been released, we utilized a previous study that reported methylated regions of seven different brain regions of the crab‐eating macaque.^13^ Within the 1310 methylated regions reported,^13^ a total of 2844 high‐confidence CpG probes were included. Comparison of DNA methylation levels revealed high consistency with different brain regions (*R* = 0.709 for the hippocampus to 0.743 for the inferior temporal lobe, average *R* for the seven brain regions was 0.733) (Figure 2). Similarly, both type I and type II probes also showed consistency, with average *R* values = .788 and .691, respectively. Because we utilized the published dataset of different brain regions of different macaques, and we did not perform validation experiments using the same brain region of same macaques, correlations remained the modest levels.

We provided high‐confidence Illumina EPIC array probes that can be used in the crab‐eating macaque genome. Using the well‐established, low‐cost platform, epigenomic research related to neuropsychiatric disorders will be facilitated in the crab‐eating macaque. In particular, given the high conservation with the human genome in each array probe, the probes can be readily used for interspecies comparisons. In this study, we used the occipital lobes, however, other tissues such as blood can be applied similarly, as we demonstrated previously in other primate.^9^

The limitations of DNA methylation assay using the methylation array are (a) it can analyze a limited number of the CpGs, compared to other approaches, such as whole‐genome bisulfite sequencing, (b) it cannot discriminate the different cytosine modifications solely, such as methylcytosine and hydroxymethyl cytosine, and (c) its quantitative data can be affected the presence of the SNPs on the array probes. It should be noted that a lack of polymorphism information in nonhuman primates might cause false methylation signals due to hybridization artifacts (Nakachi et al, unpublished data). Further refinement of the high‐confidence probes awaits the accumulation of more detailed genomic information on the crab‐eating macaque.

## CONFLICT OF INTEREST

The authors declare no conflicts of interest.

## AUTHOR CONTRIBUTIONS

TM, MB, and K. Iwamoto designed the research. K. Ishii and TM performed the experiment. YN analyzed the data. YN, MB, TM, and K. Iwamoto prepared the manuscript.

## Supporting information

Tab S1

## Data Availability

Raw data have been deposited in the Gene Expression Omnibus (https://www.ncbi.nlm.nih.gov/geo/) and are accessible through GSE154078^14^.
